# 85% internal quantum efficiency of 280-nm AlGaN multiple quantum wells by defect engineering

**DOI:** 10.1038/s41598-017-14825-8

**Published:** 2017-10-31

**Authors:** Tzu-Yu Wang, Chi-Tsung Tasi, Chia-Feng Lin, Dong-Sing Wuu

**Affiliations:** Department of Materials Science and Engineering, National Chung Hsing University, Taichung, 40227 Taiwan, R.O.C.

## Abstract

In this study, high internal-quantum-efficiency (IQE) AlGaN multiple quantum wells (MQWs) were successfully demonstrated on low-defect-density AlN templates with nano-patterned sapphire substrates. These templates consisted of AlN structures with 0∼30 periods superlattices (SLs) by alternating high (100) and low (25) V/III ratios under a low growth temperature (1130 °C). Compared to conventional high crystal-quality AlN epilayers achieved at temperatures ≥1300 °C, lower thermal budget can reduce the production cost and wafer warpage. Via optimization of the SL period, the AlN crystallinity was systematically improved. Strong dependence of SL period number on the X-ray full-width-at-half-maximum (FWHM) of the AlN epilayer was observed. The AlN template with 20-period SLs exhibited the lowest FWHM values for (0002) and (10ī2), namely 331 and 652 arcsec, respectively, as well as an ultra-low etching pit density of 1 × 10^5^ cm^−2^. The relative IQE of 280 nm AlGaN MQWs exhibited a dramatically increase from 22.8% to 85% when the inserted SL increased from 0 to 20 periods. It has hardly ever been reported for the AlGaN MQW sample. The results indicate that the engineered AlN templates have high potential applications in deep ultraviolet light emitters.

## Introduction

Recently, deep-ultraviolet light-emitting-diodes (DUV-LEDs) have attracted attention because of their wide range of potential applications in food disinfection and air/water purification, as well as in the sensing of gases (e.g., SO_2_ and NO_x_)^1^. Wide band gap materials such as AlN (6.2 eV) and Al_x_Ga_1−x_N (3.4–6.2 eV) are used to promote the epitaxial growth of the DUV-LED structure by using metalorganic chemical vapor deposition (MOCVD). Generally, sapphire substrates are chosen as epitaxial substrates for the fabrication of DUV-LEDs because of their low cost and DUV transparency. To construct high-performance devices, AlN-based epilayers require a low defect density and smooth surface. Unfortunately, a mismatch between the high lattice constants and thermal expansion coefficients of AlN and sapphire leads to an abundant dislocation density (~10^10^ to 10^11^ cm^−2^)^2^. These dislocation defects lead to the formation of a non-radiative recombination center, resulting in device degradation. Meanwhile, the dislocation defects of the AlN template strongly affect the internal quantum efficiency (IQE) of multiple quantum wells (MQWs)^3^. Therefore, several researchers have proposed methods to improve the crystallinity of AlN, including the enhancement of surface migration for Al adatoms^4^, use of AlN grown at high temperatures (≥1300 °C)^5^, AlN superlattices (SLs) with the alternation of low and high temperatures as a buffer structure^6^, the use of an AlGaN/AlN SL buffer structure^7^, and an AlN epilayer grown on a nano-patterned sapphire substrate (NPSS)^8^. Besides these methods, Shatalov *et al*. have reported enhancement in the IQE of AlGaN-based MQWs by the use of an AlN template with a low dislocation density^9^. Although high crystal-quality AlN epilayers can be obtained by utilizing a high growth temperature (≥1300 °C), a lower thermal budget (<1150 °C) is expected to reduce the production cost and wafer warpage, especially for the use of larger diameter sapphire substrates. Commonly, the production cost of the heater for a higher temperature (>1300 °C) is approximately three times more than that of the conventional heater (<1200 °C). Based on the estimation, for the same chamber life and yield rate, the cost of LED wafer produced by MOCVD with the conventional heater elements could be saved at least 70% in comparison to that with the heater for a higher temperature.

In a study reported previously by our group^10^, the AlN structure with 3-period SLs achieved by the alternation of high and low V/III ratios can effectively eliminate the dislocation density for an AlN layer grown on flat sapphire under a low temperature of 1100 °C. However, the AlN crystal quality is not sufficient for application in DUV-LEDs. Therefore, to further enhance the AlN crystallinity, the V/III ratio of the AlN structure and period number (0∼30) of the SLs grown on a NPSS are systematically optimized in this study. Meanwhile, the AlGaN MQW structure with an emission wavelength of 280 nm grown on an AlN template was utilized to confirm the epitaxial quality of AlN and the feasibility of device applications. To understand the effect of the defects of the AlN template on the MQW structure, the relative IQE of the MQWs can be obtained by photoluminescence (PL) measurements under room temperature (RT) and low temperature (LT) conditions by the using the relative relationship IQE = I_RT_/I_LT_. This relationship assumes that the IQE the MQWs is 100% at LT. Previously, Hirayama *et al*. have suggested that the highest relative IQE of 86% is achieved by using In_x_Al_y_Ga_1−x−y_N/In_x_Al_z_Ga_1−x−z_N MQWs at a wavelength of 280 nm^11^. Banal *et al*. have also proposed that the use of Al_x_Ga_1−x_N/AlN MQWs with an emission wavelength of 247 nm exhibit a relative IQE of approximately 69%^12^. In this study, the 280 nm AlGaN-based MQWs grown on a low-defect-density AlN template exhibited a relative IQE as high as 85%. The result suggests that the high-IQE AlGaN MQW structure can be achieved using an engineered low-defect-density AlN template.

## Results and Discussion

**Figure 1:**
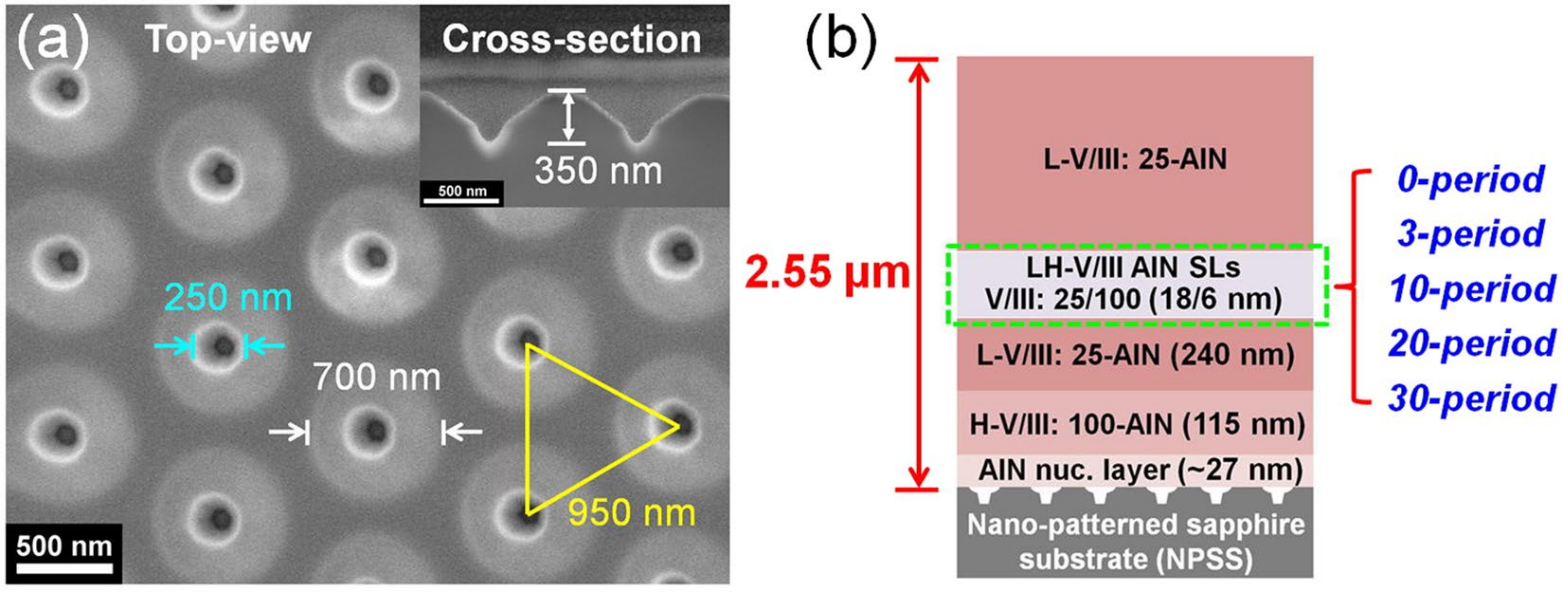
(**a**) Features of NPSS and (**b**) epitaxial structures of AlN with 0–30 period SLs.

As shown in Fig. 1(a), the NPSS exhibited funnel-shaped patterns with top and bottom diameters of 700 and 250 nm, respectively, and a depth of 350 nm. Figure 1(b) shows the AlN structures with a total thickness of 2.55 μm, the structures of which have the same buffer layers consisting of an approximately 27-nm-thick AlN nucleation layer, a 115-nm-thick high V/III (H-V/III = 100) AlN, and a 240-nm-thick low V/III (L-V/III = 25) AlN. Following the 0∼30 period LH-V/III (alternation of H- and L-V/III) AlN SLs and an L-V/III AlN top layer were deposited onto the buffer layers, respectively. Meanwhile, the thickness of the L-V/III AlN top layer increased from 2.16 to 1.45 μm with an increase in the SL period from 0 to 30.

**Figure 2:**
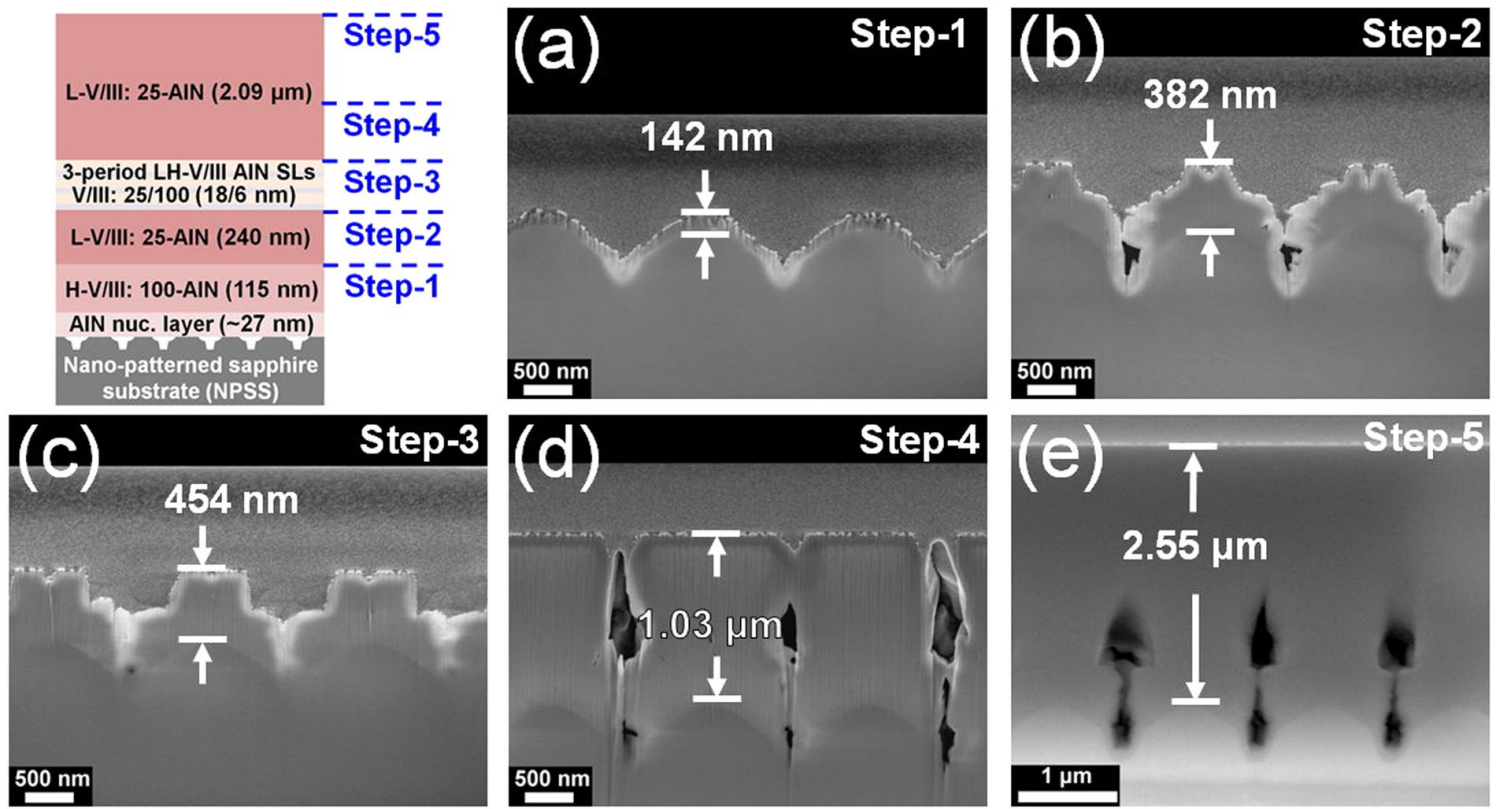
Cross-sectional FESEM images of the AlN structure grown at (**a**) step-1, (**b**) step-2, (**c**) step-3, (**d**) step-4, and (**e**) step-5.

**Figure 3:**
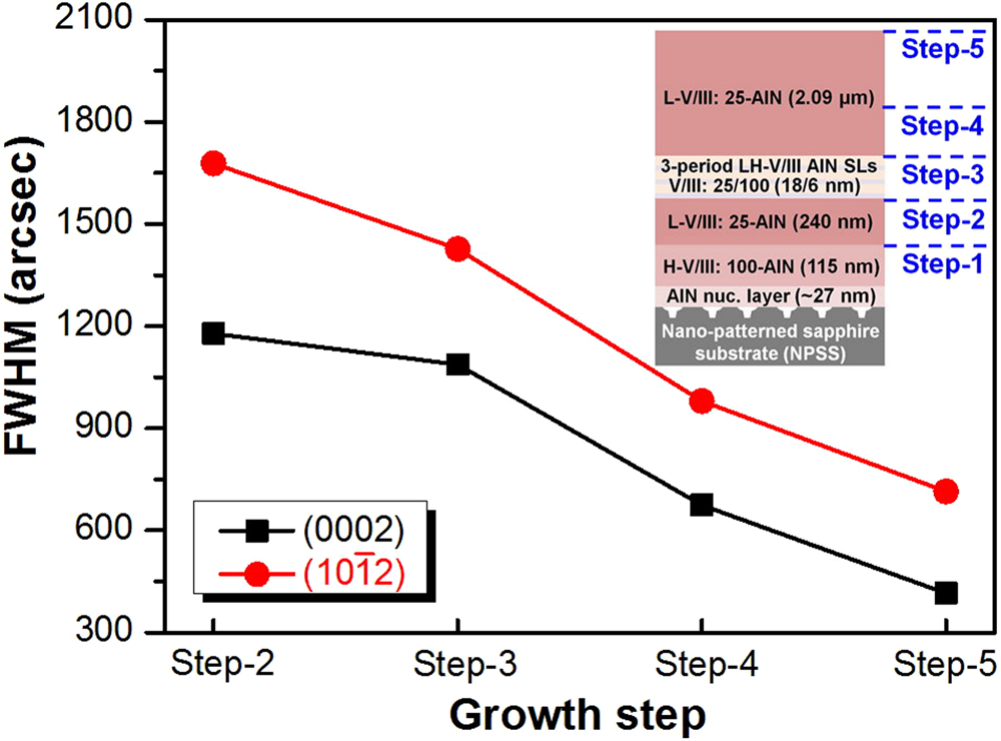
FWHM values of the AlN structure at various growth steps.

To understand the growth evolution of the AlN structure deposited on NPSS, the cross-sectional field emission scanning electron microscopy (FESEM) images of AlN with 3-period SLs recorded at various growth steps are shown in Fig. 2. At the growth step-1 (Fig. 2(a)), the AlN layer covered all of the NPSS, even the patterned regions, because of the high sticking coefficient of the Al adatoms^13^. From step-2 to step-3 (Fig. 2(b) and (c), respectively), the AlN epilayer was grown along the c-plane direction despite the increase in the lateral growth rate of the layer. At growth step-4 (Fig. 2(d)), the epilayer coalesced, and some holes were observed on the patterned regions. These results indicated that the growth evolution of the AlN epilayer is dominated by epitaxial lateral overgrowth (ELOG). Finally, the flat AlN epilayer was observed at growth step-5 (Fig. 2(e)). Notably, some key-holes were formed on the patterned regions, which could decrease the tensile stress caused by the lattice mismatch between the AlN layer and sapphire. Figure 3 shows the further evaluation of the crystallinity of the AlN epilayers in terms of the full width at half maximum (FWHM) values for (0002) and (10ī2) for the AlN structure from the growth step-2 to step-5. The (0002) and (10ī2) FWHM values decreased as a function of the growth steps. The FWHM value of (0002) decreased from 1178 to 415 arcsec, while that of (10ī2) decreased from 1678 to 714 arcsec. The (0002) FWHM corresponded to screw dislocations, while the (10ī2) FWHM corresponded to the edge and mixed dislocations^14^. In this study, the growth evolution of the epilayer was dominated by ELOG as shown in the FESEM images in Fig. 2. The decrease in both (0002) and (10ī2) FWHM values was related to the fact that ELOG can assist in the elimination of dislocations by bending or combining with each other.

**Figure 4:**
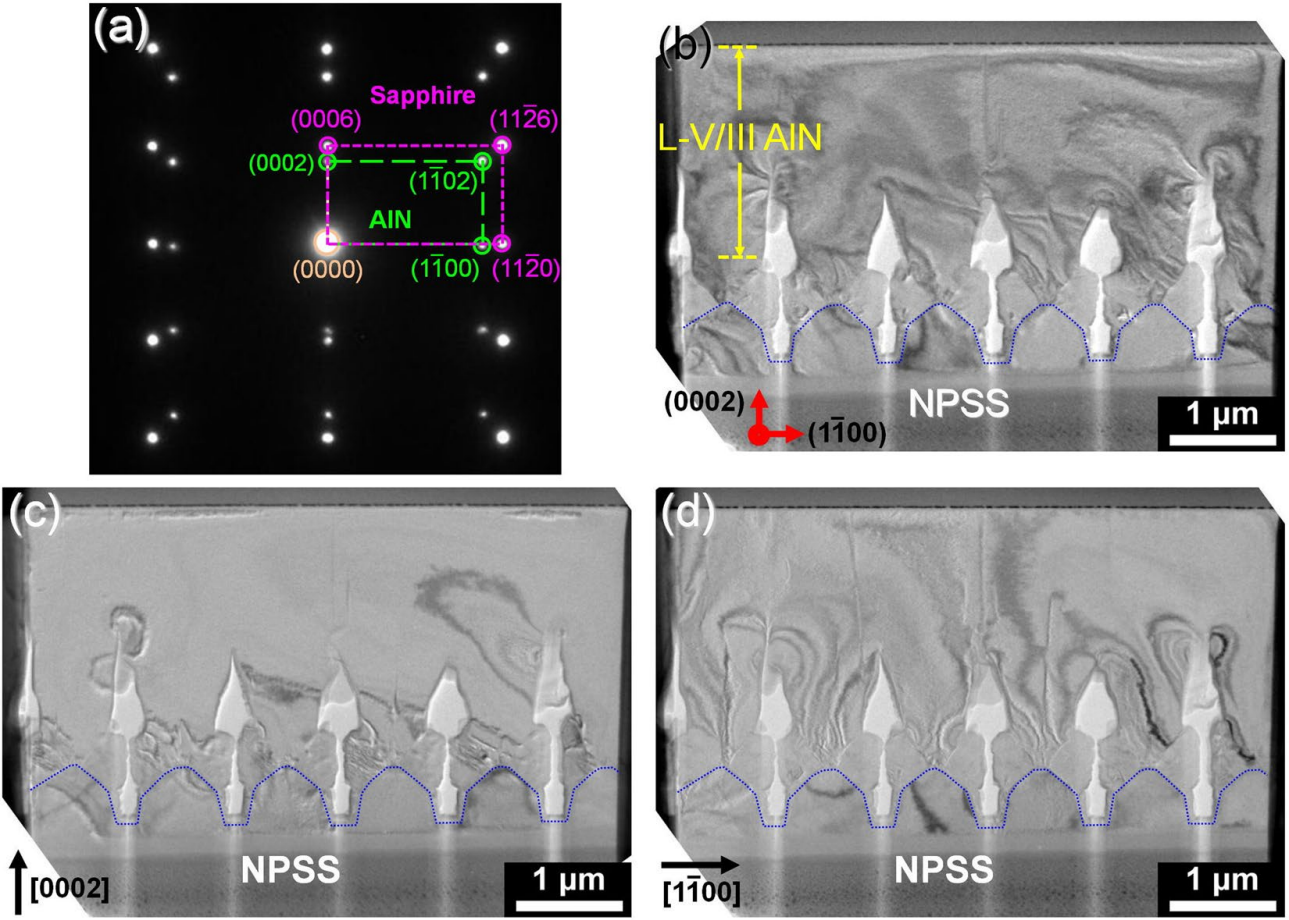
(**a**) Diffraction pattern of the AlN/sapphire interface. (**b**) Cross-sectional bright-field TEM image of the AlN structure/NPSS. TEM images with a two-beam condition along (**c**) g = 0002 and (**d**) g = 1–100.

**Figure 5:**
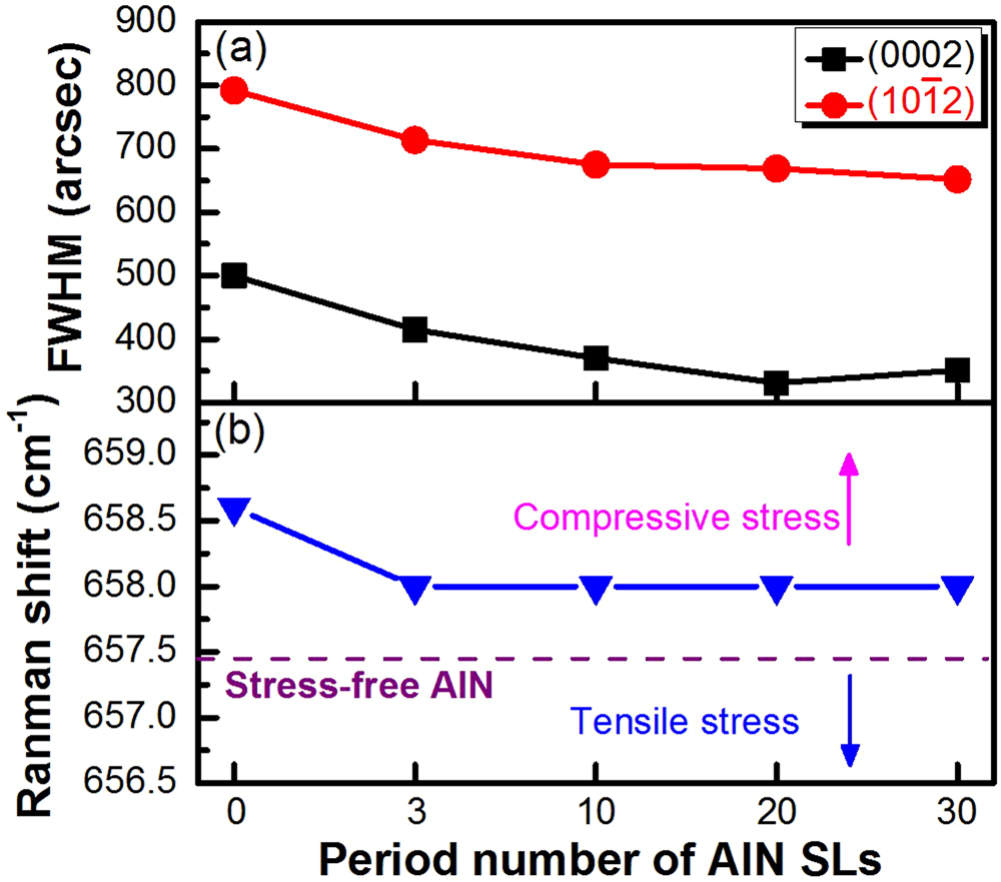
(**a**) FWHM values and (**b**) Raman shift of AlN with 0–30 period SL structures.

Figure 4 shows the epitaxial relationship and dislocation behavior of AlN grown on NPSS. The diffraction pattern at the AlN/NPSS interface (Fig. 4(a)), where the relationship is AlN (0002)||sapphire (0006) and AlN (1ī00)||sapphire (11^–^20), indicating that the AlN epilayer exhibits a preferred (0002) orientation (along the c-plane direction). In our previous research10, it can clearly indicate that the insertion of AlN SLs into the AlN epilayer (on flat sapphire) is beneficial to decrease the dislocation density. Without inserting the AlN SLs, the dislocations can easily propagate from the AlN/sapphire interface to the surface of AlN epilayer. However, after inserting the AlN SLs into the AlN epilayer, the dislocation propagation can be eliminated efficiently. This is why the insertion of AlN SLs was used during the growth of AlN epilayer on NPSS. Actually, the insertion of AlN SLs is also helpful to reduce the dislocation density of the AlN epilayer on NPSS, as discussed in Fig. 5(a). The cross-sectional bright-field transmission electron microscopy (TEM) image of the AlN (Fig. 4(b)) revealed only a few dislocations propagating to the layer surface. The total dislocation density near the layer surface was estimated to be approximately 1 × 10^8^ cm^−2^, suggesting that most dislocations are eliminated by inducing the SL structure and ELOG. The TEM images shown under a two-beam condition are utilized to distinguish the screw, edge, and mixed dislocations, as shown in Fig. 4(c) and (d). The Burgers vector (b) of the screw dislocation is b = <0001> and that of edge dislocations is b = 1/3 <1ī20>^15^. Based on the invisible criterion g·b = 0, the screw and edge dislocations were observed along g = 0002 and g = 1ī00, respectively. Meanwhile, mixed dislocations were observed at g = 0002 and g = 1ī00. Notably, the number of edge dislocations was greater than the screw dislocations near the layer surface, corresponding to the (0002) FWHM being less than the (10ī2) FWHM.

To enhance the crystal quality of the AlN structure grown on NPSS, the period number of the SLs was optimized. In Fig. 5(a), both (0002) and (10ī2) FWHM values gradually reduced with the increase in the SL period from 0 to 30. Moreover, the decrease in the FWHM values attained stability at SL period greater than 10. Before inserting the AlN SLs, the (0002) and (10ī2) FWHM values of the AlN epilayer were 500 and 792 arcsec, respectively. In the SL periods of 0–30, the lowest (0002) FWHM value of 331 arcsec and (10ī2) FWHM value of 652 arcsec were obtained by utilizing the 20-period SLs. These results indicated that the SLs can effectively assist in the elimination of dislocations. The screw and edge dislocation densities (D_screw_ and D_edge_) can be derived from the following equations: D_screw_ = β_(002)_/9b^2^_screw_ and D_edge_ = β_(102)_/9b^2^_edge_^16^. In these two equations, β_(002)_ and β_(102)_ represent the FWHM values of AlN(0002) and AlN(10ī2), respectively. Additionally, b is the Burger vector length. Here, bedge and bscrew are 0.3189 and 0.5185 nm, respectively. Via our calculations, the screw and edge dislocation densities of the AlN epilayer without inserting the AlN SLs are 5.02 × 10^8^ and 2.61 × 10^10^ cm^−2^, respectively. With the insertion of the 20-period SLs, these two dislocation densities can be reduced to 2.20 × 10^8^ and 2.01 × 10^10^ cm^−2^, respectively. In comparison to the dislocation density (1 × 10^8^ cm^−2^) from the TEM image (Fig. 4(b)), these calculated dislocation densities are higher. This is attributed that the estimation of dislocation density via the TEM result is observed near the epilayer surface. It can be found that the reduction of screw dislocation density is more obvious when the technique of SLs combined with NPSS is used. In addition to AlN crystallinity, the stress management of AlN is crucial for the fabrication of DUV-LEDs. Hence, the residual stress of AlN with 0∼30 period SL structures is estimated by Raman measurement (Fig. 5(b)). The frequency of the E_2_ high phonon mode was located at 658.6 cm^−1^ for AlN with 0-period SLs and at 658 cm^−1^ for AlN with 3∼30 period SL structures. For stress-free AlN^17,18^, the frequency was located at 657.4 cm^−1^. Notably, the AlN epilayers with 0∼30 period SL structures exhibited a higher frequency compared to the stress-free frequency, which is indicative of compressive stress. Moreover, the in-plane compressive stress (σ) of AlN can be evaluated by the following equation^17,18^:

$$C{\rm{\sigma }}={{\rm{\omega }}}_{{\rm{E2}}(\mathrm{high})}-{{\rm{\omega }}}_{0}$$

Here, C is the biaxial strain coefficient (3 cm^−1^/GPa)^19^, and ω_E2 (high)_ and ω_0_ are the frequencies of the E_2_ high phonon mode for AlN with SL structures and stress-free AlN, respectively. Hence, the compressive stress of AlN with 0 period and 3∼30 period SL structures are 0.4 and 0.2 GPa, respectively. This result indicated that the compressive stress state is related to the existence of SLs on the patterned regions. A similar result has been reported by Dong *et al*.^17^, wherein the AlN epilayer with a frequency of 658.7 cm^−1^ and a compressive stress of 0.43 GPa are obtained for AlN grown on NPSS at a temperature of 1200 °C. In this study, the AlN with SL structures grown under a low temperature of 1130 °C revealed a lower compressive stress.

**Figure 6:**
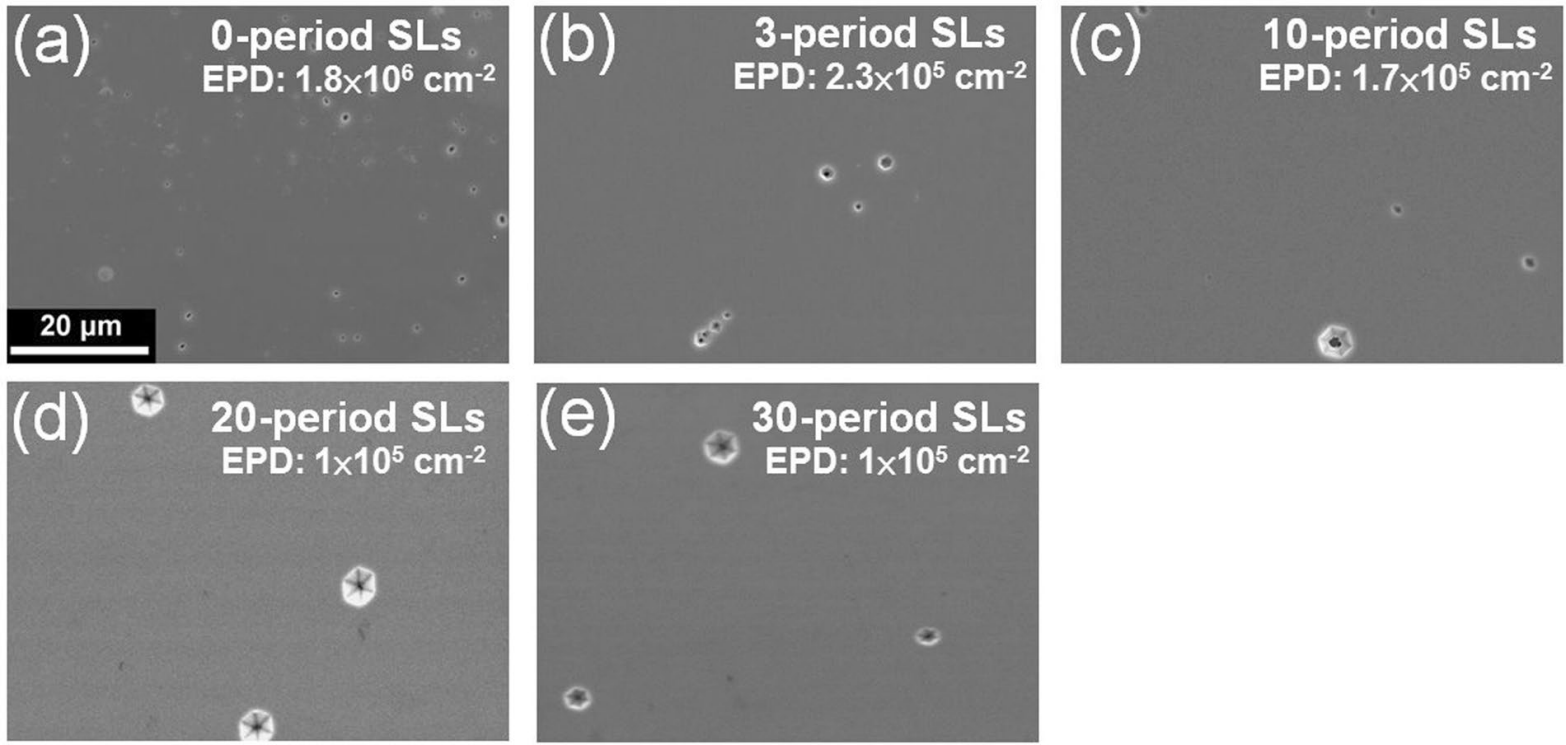
Surface morphology images of AlN with (**a**) 0, (**b**) 3, (**c**) 10, (**d**) 20, and (**e**) 30 period SL structures after etching in a KOH solution.

To realize the density of the dislocations that have propagated to the epilayer surface, the etching pit density (EPD) of AlN with SL periods 0∼30 was estimated (Fig. 6). After etching in the KOH solution, the difference in the etching rates of the screw, edge, and mixed dislocations led to etching pits with different pit sizes. Kitagawa *et al*. have suggested that the smallest etching pits correspond to edge-type dislocations, while the largest hexagonal pits correspond to screw or mixed type dislocations^20^. In this study, edge-type dislocations decreased with the increase in the SL period from 0 to 30 owing to dislocation elimination (Fig. 6(a–e)). Overall, the EPD decreased from 1.8 × 10^6^ to 1 × 10^5^ cm^−2^ with the increase in the SL period from 0 to 30. In contrast to the EPD of 2 × 10^6^ cm^−2^ reported by Kim *et al*.^7^ and the EPD of 1 × 10^6^ cm^−2^ reported by Tran *et al*.^21^, the AlN with 20∼30 period SLs revealed an ultra-low EPD value of 1 × 10^5^ cm^−2^ in this study, indicating that the increase in the SL period effectively assists in the decrease of the dislocation density. This result corresponds with the FWHM values of AlN along (0002) and (10–12). To achieve a high relative IQE of the AlGaN MQW structure, the AlN with 20-period SLs was utilized as a growth template.

**Figure 7:**
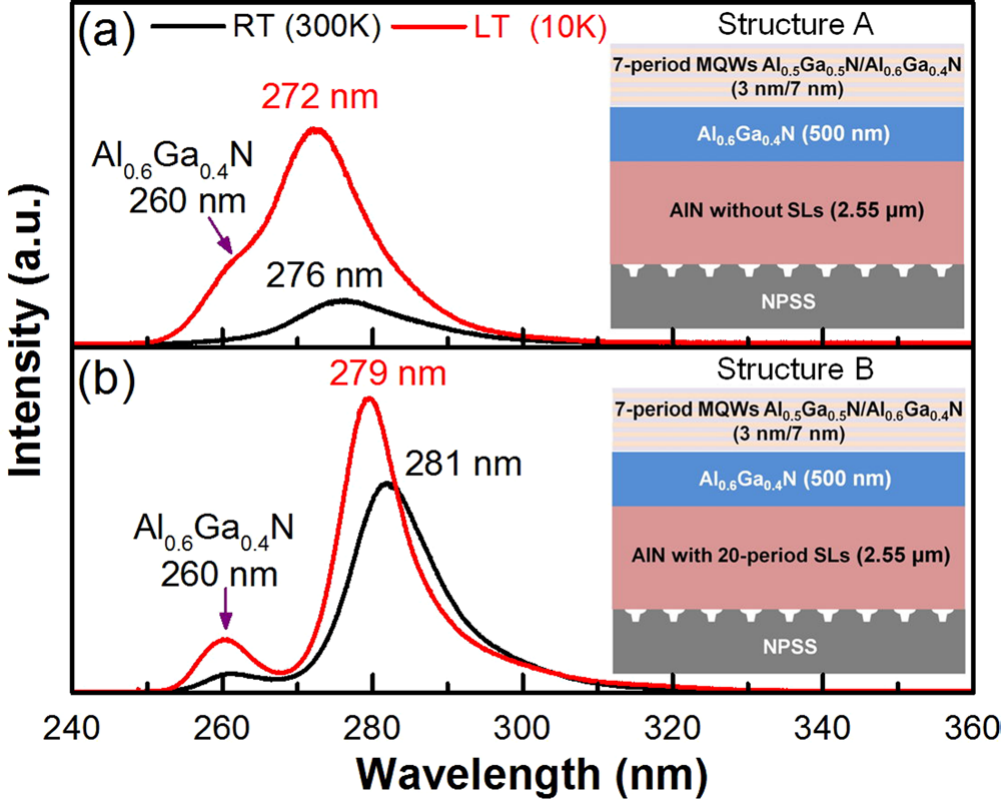
PL spectra of (**a**) structure A and (**b**) structure B under RT and LT.

To confirm the defect density of the AlN template on the relative IQE of AlGaN-based MQWs, 280-nm-MQW structures were prepared on AlN both without and with 20-period SLs, which are denoted as structures A and B, respectively. For structure A (shown in Fig. 7(a)), the emission wavelength of the MQWs was observed at 276 nm for RT and 272 nm for LT (10 K). A clear shoulder peak at 260 nm for the LT was caused by the Al_0.6_Ga_0.4_N barrier layer and buffer layer. If the IQE of the MQWs under the LT condition was assumed to be 100%, the relative IQE of structure A at RT can be evaluated as 22.4%. In contrast to structure A, structure B exhibited narrower emission peaks at 281 nm for RT and 279 nm for LT (Fig. 7(b)). The relative IQE of structure B was estimated as 85%, indicating that the 280-nm-MQW structure grown on a low-defect-density AlN template effectively enhances the relative IQE. By comparing the RT-PL characteristics of AlGaN MQWs on AlGaN/AlN templates without and with the AlN SLs (Fig. 7(a) and (b)), we can observe that the main emission wavelengths of these two MQWs were centered at 276 and 281 nm, respectively. The composition-pulling effect can be used to realize the result. Without inserting the AlN SLs, there existed a relatively larger compressive stress in the wells. This would result in the composition tendency of AlGaN wells towards higher Al content to minimize the lattice mismatch^22^. However, as the AlN SLs were inserted into the AlN epilayer, the compressive stress in the wells became smaller, leading to the lower Al content in AlGaN wells. This is why the AlGaN MQWs on AlGaN/AlN template without inserting the AlN SLs have a shorter emission wavelength. In addition, the other interesting phenomenon was also observed in PL results. Comparing the main RT-PL peaks of these two structures, the AlGaN MQWs on AlGaN/AlN template without inserting the AlN SLs possessed an apparent larger FWHM value (149.9 nm) than that with inserting the AlN SLs (14.1 nm). There could be two reasons to explain it. The first reason is the formation of quantum-confined Stark effect (QCSE) in the structure without the AlN SLs, which causes the band tilt^23,24^. Second, due to the higher defect density of AlN epilayer without the insertion of AlN SLs, the quality of the AlGaN MQWs was lower. Thus, the FWHM value of RT-PL peak for the structure A was apparently large. In general, the QCSE is related to the red-shift of emission peak. However, compared to the PL characteristic of the structure B (with inserting the AlN SLs), the blue-shift phenomenon occurred in the structure A. We can speculate that this shift of emission peak is mainly dominated by the composition-pulling effect. Besides, the differences between emission peaks measured at RT and LT for the AlGaN MQWs on AlGaN/AlN templates without and with inserting the AlN SLs were measured to be 4 and 2 nm, respectively. The larger shift of PL peak in the structure A is possible owing to its higher compressive stress in the wells measured at RT. On the other hand, the relative IQE values obtained from previous studies as well as in this study are summarized in Table 1. As shown in Table 1, Hirayama *et al*. have obtained a high relative IQE of 86% using the In_x_Al_y_Ga_1−x−y_N/In_x_Al_z_Ga_1−x−z_N MQWs. For the AlGaN-based MQWs, the relative IQE of the listed research results is shown to be around 43–69%. For these previous studies, the AlN templates were prepared at a high growth temperature (≥1250 °C). Herein, a high relative IQE of 85% for 280 nm AlGaN-based MQWs was achieved by using an AlN template grown under a low growth temperature (1130 °C) and it has hardly ever been reported for AlGaN one. Therefore, the low-EPD AlN templates designed by defect reduction engineering demonstrate high potential for high-IQE DUV-LEDs applications.

**Table 1:** Summary of wavelength, MQWs structure, and relative IQE from previous research results and this study.

Wavelength	MQW structure (Well/Barrier)	Condition of PL measurement at RT/LT	Relative IQE I_RT_/I_LT_	Author^ref.^
247 nm	Al_x_Ga_1−x_N/AlN	300 K/8.5 K	69%	Banal *et al*.^12^
250 nm	Al_0.7_Ga_0.3_N/AlN	285 K/12 K	50%	Bhattacharyya *et al*.^25^
278 nm	Al_x_Ga_1−x_N/Al_y_Ga_1−y_N	300 K/14 K	55%	Shatalov *et al*.^9^
280 nm	Al_x_Ga_1−x_N/Al_y_Ga_1−y_N	300 K/77 K	50%	Hirayama *et al*.^11^
	In_x_Al_y_Ga_1−x−y_N/In_x_Al_z_Ga_1−x−z_N		86%	
283 nm	Al_0.4_Ga_0.6_N/Al_0.5_Ga_0.5_N	300 K/10 K	43%	Dong *et al*.^17^
281 nm	Al_0.5_Ga_0.5_N/Al_0.6_Ga_0.4_N	300 K/12 K	85%	NCHU

## Conclusions

The low-defect-density AlN templates were successfully fabricated under a low growth temperature (1130 °C) to achieve high-IQE MQWs. From the FESEM analysis of the growth evolution of AlN with 3-period SLs grown on NPSS, the growth of the AlN layers was dominated by ELOG. Meanwhile, some key-holes were observed upon the pattern regions. These key-holes could be relaxed the tensile stress, related to the lattice mismatch between AlN and sapphire. Besides, the (0002) and (10ī2) FWHM values decreased with the increase in the growth step because of the elimination of dislocations. From the TEM analysis, the ELOG and induction of SLs can assist in the elimination of most dislocations, with only a few of the dislocations propagating to the layer surface. As a result, the total dislocation density of AlN with 3-period SLs is estimated to be approximately 1 × 10^8^ cm^−2^. The further enhancement of the AlN crystal quality was achieved by the increase in the SL period (0∼30), and the AlN structure with 20-period SLs exhibited the lowest (0002) FWHM value of 331 arcsec and (10ī2) FWHM value of 652 arcsec, as well as an ultra-low EPD of 1 × 10^5^ cm^−2^. Therefore, the AlN structure with 20-period SLs is selected as a growth template for preparing the AlGaN MQWs with an emission wavelength of 280 nm. Notably, the relative IQE dramatically enhanced by approximately four times (from 22.8% to 85%) for the growth of 280-nm-MQWs on the AlN template with 20-period SLs. These results exhibit that the presented the AlN templates by defect engineering can provide an alternate approach in developing high-efficient DUV-LEDs.

## Methods

In this study, the AlN epitaxial structures were grown on 2-inch NPSS by utilizing Aixtron MOCVD. The growth temperature, pressure, and V/III ratio of the AlN nucleation layer were 1000 °C, 100 mbar, and 800, respectively. Other layers were grown at 1130 °C under a pressure of 100 mbar. The crystallinity of all of the AlN epilayers was measured by X-ray diffraction. The growth evolution and dislocation behavior of the AlN epilayers were observed by FESEM and TEM. The residual stress of the AlN layers was estimated by Raman measurement. The EPD of AlN was obtained by etching in a 45 wt% KOH solution at 75 °C for 4.5 min. To further verify the effect of the defect density of the AlN template on the relative IQE of 280-nm-MQWs, an Al_0.6_Ga_0.4_N buffer layer with a thickness of 500 nm and 7-period Al_0.5_Ga_0.5_N (3 nm)/Al_0.6_Ga_0.4_N (7 nm) MQWs were prepared on AlN both with and without 20-period LH-V/III AlN SLs. The relative IQE of 280-nm-MQWs was estimated by PL measurement with an excitation laser of 213 nm under RT and LT, following the relative formula IQE = I_RT_/I_LT_.
